# Differences in Mortality and Sepsis-Associated Organ Dysfunction between Surgical and Non-Surgical Sepsis Patients

**DOI:** 10.3390/biomedicines11082233

**Published:** 2023-08-09

**Authors:** Caspar Mewes, Julius Runzheimer, Carolin Böhnke, Benedikt Büttner, Marcus Nemeth, José Hinz, Michael Quintel, Ashham Mansur

**Affiliations:** 1 Department of Anesthesiology, University Medical Center Goettingen, 37075 Goettingen, Germany; carolin.bhnke@stud.uni-goettingen.de (C.B.); benedikt.buettner@med.uni-goettingen.de (B.B.); marcus.nemeth@med.uni-goettingen.de (M.N.); mquintel@med.uni-goettingen.de (M.Q.); ashham.mansur@med.uni-goettingen.de (A.M.); 2 Center of Anesthesiology and Intensive Care Medicine, University Medical Center Hamburg-Eppendorf, 20251 Hamburg, Germany; 3 Department of Neurology and Neurophysiology, University Medical Center Freiburg, 79106 Freiburg, Germany; 4 Department of Anesthesiology and Intensive Care Medicine, Klinikum Region Hannover, 30459 Hannover, Germany; jose.hinz@krh.eu; 5 Department of Anesthesiology, Asklepios Hospitals Schildautal, 38723 Seesen, Germany

**Keywords:** sepsis, surgical sepsis, non-surgical sepsis, mortality, organ dysfunction

## Abstract

(1) Background: Patients with sepsis following surgical intervention may exhibit fundamental distinctions from those experiencing sepsis without prior surgery. Despite the potential clinical importance of distinguishing these two sepsis subpopulations, dissimilarities, particularly in outcome, between surgical and non-surgical patients have been subject to limited scientific investigations in the existing literature. This study aimed to investigate the differences in mortality and sepsis-associated organ dysfunction between these two groups. (2) Methods: A retrospective analysis was conducted using data from a large cohort of prospectively enrolled patients with sepsis (n = 737) admitted to three intensive care units at University Medical Center Goettingen; patients were categorized into surgical (n = 582) and non-surgical sepsis groups (n = 155). The primary outcomes assessed were 28- and 90-day mortality rates, and secondary endpoints were multiple clinical parameters and measures of sepsis-associated organ dysfunction. (3) Results: Non-surgical patients presented a significantly higher 90-day mortality (37%) compared to surgical sepsis patients (30%, *p* = 0.0457). Moreover, the non-surgical sepsis group exhibited increased sepsis-associated organ dysfunction, as evidenced by higher average SOFA scores (*p* < 0.001), elevated levels of serum Procalcitonin (*p* = 0.0102), and a higher utilization of organ replacement therapies such as ventilation (p < 0.001), vasopressor treatment (*p* < 0.001), and renal replacement therapy (*p* = 0.0364). Additionally, non-surgical sepsis patients had higher organ-specific SOFA respiratory (*p* < 0.001), cardiovascular (*p* < 0.001), renal (*p* < 0.001), coagulation (0.0335), and central nervous system (*p* = 0.0206) subscores. (4) Conclusions: These results suggested that patients with non-surgical sepsis may face distinct challenges and a higher risk of adverse outcomes compared to patients with sepsis following surgical intervention. These findings have important implications for clinical decision-making, patient management, and resource allocation in sepsis care.

## 1. Introduction

Sepsis is a severe medical condition characterized by infection-induced dysregulated host immune response and subsequent organ dysfunction [1]. It remains a major global health concern, contributing to substantial morbidity and mortality rates [2,3,4]. While extensive research has focused on understanding the impact of sepsis on outcomes, limited research has directly compared the outcomes between surgical and non-surgical patients.

As sepsis is a prevalent and multifaceted medical condition encountered across various departments of the hospital, sepsis patients encompass a highly heterogeneous patient cohort [5,6]. Patients, for instance, vary inter-individually by the involved pathogens, the prior site of infection, preexisting comorbidities, or status of immune response, whereas therapy regimes remain limited to untailored supportive care [6].

The subgroup of patients with postoperative sepsis represents a distinct subset within this broader sepsis population. The nature of surgical intervention introduces unique factors, such as surgical site infections, perioperative stress, and alterations in immune response that contribute to the complexity and clinical characteristics of postoperative sepsis patients [7,8,9].

In contrast, patients with sepsis unrelated to surgery often present with severe underlying medical conditions that can exacerbate the severity of their septic condition [10]. These comorbidities, which may include chronic respiratory, cardiovascular, renal, and neurologic diseases, can further compromise their immune response and increase the risk of adverse outcomes [11,12].

Investigating the differences in mortality and sepsis-associated organ dysfunction between postoperative sepsis and non-interventional sepsis patients is crucial for understanding the distinct challenges faced by each group. This retrospective analysis of data from a large cohort of prospectively enrolled patients with sepsis aimed to reveal significant differences in mortality and sepsis-associated organ disfunction between surgical and non-surgical patients with sepsis. 

## 2. Materials and Methods

The present study was performed at the University Medical Center Goettingen, Ger- many, between 2012 and 2019. All investigations and study protocols were approved under the ethical project identification code 1/15/12 by the responsible institutional ethics committee of the University of Goettingen. The study was performed in accordance with the provisions of the Helsinki Declaration. Written informed consent was obtained from all patients or their legal representatives.

The study adhered to and followed the STROBE (Strengthening the Reporting of Observational Studies in Epidemiology) guideline regulations throughout the investigation [13].

### 2.1. Patient Enrollment 

This study comprised a secondary analysis of data from a previous prospective, observational cohort study, which aimed to investigate the association of genetic and clinical data with mortality and patient-centered clinical outcomes in individuals diagnosed with sepsis and septic shock. The study population consisted of 737 patients with clinically defined sepsis that were enrolled from three anesthesiologic ICUs at University Medical Center Goettingen, Germany, between 2012 and 2019. The cohort comprised individuals from various settings, including patients with severe infections in the community, patients with extended medical treatment on general wards (e.g., pneumonia, NSTEMI, and coronary heart disease), and postoperative patients from diverse surgical disciplines. The surgical cases involved a wide range of conditions, including general surgery (cholecystitis, esophageal rupture, mediastinitis, perforated appendicitis, necrotizing pancreatitis, ileus, etc.), trauma surgery (polytrauma and periprosthetic infections), thoracic, cardiac, and vascular surgery (aortocoronary bypass, valve surgeries, etc.), neurosurgery (intracranial bleeding, spondylodiscitis, etc.), and ear, nose, and throat (ENT) surgery (retropharyngeal abscess, tongue base cancer, and hypopharynx cancer).

Continuous patient enrollment adhered to the most up-to-date internationally recognized consensus guidelines and definitions for sepsis and septic shock at this time [1,14]. Eligible patients were identified and monitored for a maximum duration of 28 days, unless they were discharged or experienced mortality prior to that period. The assessment of mortality rates was conducted through telephone follow-up or written request from the local registry, at the 28- and 90-day timepoints. 

Previously described exclusion criteria were applied [15,16,17,18,19,20,21,22]:

- Age below 18 years;
- Pregnancy or breastfeeding;
- Immunosuppressive drugs and/or chemotherapy within six months prior to enrollment;
- History of myocardial infarction within six weeks before recruitment;
- New York Heart Association stage IV chronic heart failure;
- Human immunodeficiency virus (HIV) infection and/or hepatitis B/C infection;
- End-stage incurable disease;
- Persistent vegetative state (apallic syndrome);
- “Do Not Treat” or “Do Not Resuscitate” order;
- Participation in interventional studies;
- Family member of a study-site employee.

Eligible patients were categorized according to their recent surgical history into surgical patients (including elective and emergency surgery) and non-surgical patients that did not undergo any type of surgical intervention. Surgical history included cardiac and non-cardiac surgery, including neurosurgery.

### 2.2. Data Collection 

All clinical and patient baseline data were extracted from the electronic patient record system, specifically the IntelliSpace Critical Care and Anesthesia (ICCA) software developed by Philips Healthcare, Andover, MA, USA. The data collection was performed using standardized clinical report forms (CRFs).

Upon enrollment, pertinent baseline characteristics were collected, including comorbidities, preexisting medication, as well as the initial Sequential Organ Failure Assessment (SOFA) and Acute Physiology and Chronic Health Evaluation (APACHE II) scores. 

Patients were subsequently monitored for a period of 90 days, with 28- and 90-day mortality being recorded as the primary outcome parameters.

Throughout the initial 28 days following sepsis onset, significant clinical data were generated including SOFA score-relevant organ dysfunction variables, information about organ replacement therapies, and inflammatory, kidney, and liver values.

### 2.3. Statistical Analysis 

For the statistical analysis of the present study STATISTICA 13 software (version 13.0, StatSoft, Tulsa, OK, USA) was used. A *p*-value < 0.05 was considered statistically significant. For the Kaplan–Meier survival analysis, a log-rank test was applied. Continuous variables were analyzed using the Mann–Whitney U-Test, whereas categorical variables were assessed using either Pearson’s chi-square-test or two-sided Fisher’s exact test, if applicable. Categorical variables are presented as absolute numbers or percentages and continuous variables are presented as mean ± standard deviation or median and interquartile ranges, respectively. 

To adjust for the effect of confounders on survival, multivariate Cox regression analyses were conducted. They were divided into one analysis involving relevant epidemiologic baseline characteristics and parameters that differed significantly between the two groups at baseline and another analysis that also included time-varying covariates that differed during observation.

## 3. Results

### 3.1. Demographics and Patient Baseline Characteristics

For this study, a total of 737 patients were enrolled. At baseline, the average age of the study population was 63 years. Two thirds (66%) of them were male, and the average Body Mass Index (BMI) was 28. More than half of the patients (51%) were in septic shock during the observation. 

The patients were categorized into a surgical sepsis group (n = 582) and a non-surgical sepsis group (n = 155). At baseline, significant differences between the two groups were observed in terms of BMI (27 ± 6 vs. 30 ± 10 kg/m^2^, *p* = 0.0354), initial severity scores, the prevalence of common comorbidities, preexisting medication, and the primary site of infection (*p* < 0.001).

Non-surgical patients exhibited significantly higher day 1 SOFA (11 ± 4 vs. 9 ± 4, *p* < 0.001) and APACHE II scores (23 ± 7 vs. 21 ± 7, *p* < 0.001) compared to surgical sepsis patients.

The prevalence of chronic obstructive pulmonary disease (COPD; 21% vs. 13%, *p* = 0.0208) and bronchial asthma (5% vs. 2%, *p* = 0.0136) was higher in the non-surgical cohort, whereas cancer was more frequent in the surgical cohort (16% vs. 7%, *p* = 0.0062).

Regarding the preexisting medication, surgical patients had a higher rate of statin use compared to non-surgical patients (25% vs. 17%, *p* = 0.0268).

All presented results can be obtained from Table 1.

### 3.2. Survival Analysis 

**Figure 1:**
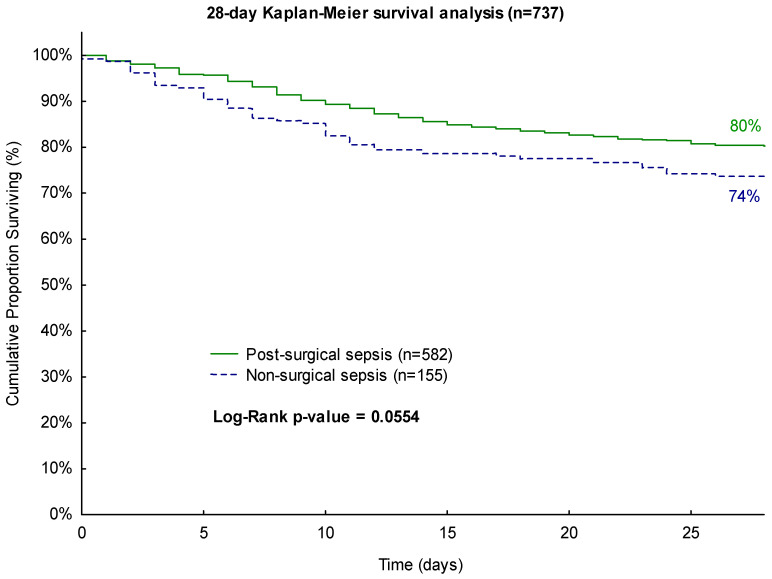
Kaplan–Meier 28-day survival analysis with regard to surgical history.

The conducted Kaplan–Meier survival analyses showed similar trends. The survival analysis conducted for the 28-day observation period indicated a higher survival rate for patients who underwent surgery (80% vs. 74%). However, this result did not reach statistical significance (*p* = 0.0554, Figure 1). 

**Figure 2:**
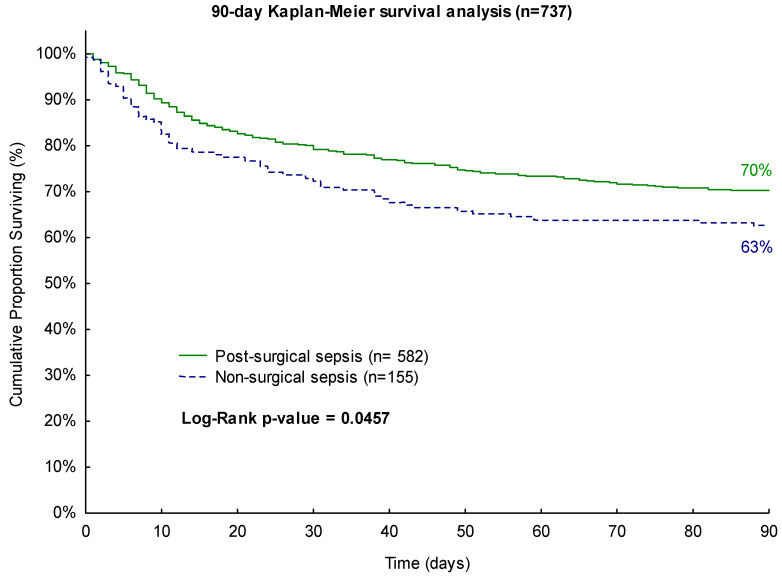
Kaplan–Meier 90-day survival analysis with regard to surgical history.

For the 90-day observation period, surgical sepsis patients exhibited a favorable survival rate of 70% compared to non-surgical patients with a survival rate of only 63% (*p* = 0.0457, Figure 2).

### 3.3. Disease Severity Analysis 

Non-surgical patients demonstrated a significantly higher sepsis disease severity compared to surgical patients, as indicated by several objective measures (Table 2). This included higher average SOFA scores (*p* < 0.001), a longer duration in septic shock (*p* = 0,0285), elevated levels of serum Procalcitonin (*p* = 0.0102), and a greater utilization of organ replacement therapies, such as ventilation (*p* < 0.001), vasopressor treatment (*p* < 0.001), and dialysis (*p* = 0.0364). 

Additionally, non-surgical sepsis patients exhibited higher organ-specific SOFA respiratory (*p* < 0.001), cardiovascular (*p* < 0.001), renal (*p* < 0.001), coagulation (*p* = 0.0335) and central nervous system (*p* = 0.0206) subscores.

Regarding their renal functions, non-surgical patients presented higher serum creatinine values (*p* < 0.001), lower urin outputs per day (*p* < 0.001), and per kg body weight per hour (*p* < 0.001).

With an average of 41 compared to 32 days, the hospital length of stay was longer in surgical patients compared to non-surgical sepsis patients (*p* < 0.001).

### 3.4. Multivariate Cox Proportional Hazards Regression Analysis 

The multivariate Cox regression analyses for the 90-day and 28-day mortality, including variables that varied at baseline, revealed that age (*p* < 0.001) and the initial SOFA score (*p* < 0.001) significantly impacted both 90-day and 28-day mortality (Table 3). BMI only had a significant impact on 28-day mortality (HR = 0.97; 95%-CI: 0.94–1; *p* = 0.0243) but did not reach statistical significance for 90-day mortality. History of cancer significantly affected 90-day mortality (HR = 1.44; 95%-CI = 1.01–2.03; *p* = 0.0418).

Prior surgical intervention emerged as a significant prognostic variable for 90-day mortality (*p* = 0.0428) with an adjusted hazard ration of 0.73 (95%-CI = 0.53–0.99).

The multivariate Cox regression model, including parameters that differed between the two investigated groups at baseline as well as time-varying covariates that differed during observation, revealed the following results (Table 4): Age (*p* < 0.001), SOFA day 1 (*p* < 0.001), medical history of cancer (90-day mortality: *p* < 0.001, 28-day mortality: 0.0229), hospital length of stay (*p* < 0.001), SOFA cardiovascular subscore (90-day mortality: *p* < 0.001, 28-day mortality: 0.0092), SOFA central nervous system subscore (*p* < 0.001), SOFA renal score (90-day mortality: *p* = 0.0032, 28-day mortality: 0.018), and the fraction of dialysis days during observation days (90-day mortality: *p* < 0.001, 28-day mortality: 0.0143) significantly impacted either 28- and 90-day mortality. The SOFA respiratory subscore (*p* = 0.022) only had a significant impact on 90-day mortality, whereas BMI (*p* = 0.0421) and days in septic shock (*p* = 0.0494) were shown to be relevant prognostic factors for 28-day mortality.

Surgical intervention was neither shown to be a relevant predictor for the 90-day mortality (HR = 1.08; 95%-CI = 0.78–1.5; *p* = 0.6321) nor the 28-day mortality (HR = 0.98; 95%-CI = 0.64–1.47; *p* = 0.9052) in the multivariate analysis, including time-varying covariates.

## 4. Discussion

This study aimed to investigate differences in mortality and sepsis-associated organ disfunction between surgical and non-surgical patients with sepsis. 

The primary finding of this investigation was that non-surgical patients had a significantly higher 90-day mortality rate (37%) compared to surgical sepsis patients (30%, *p* = 0.0457). After adjusting for relevant confounders in the multivariate Cox regression analysis, surgical intervention remained an independent prognostic variable for 90-day survival (HR 0.73, 95%-CI 0.53–0.99, *p* = 0.0428), considering variables that varied between the two investigated groups at baseline. This result could not be replicated in the following multivariate analysis that also included time-varying covariates that differed during observation. This result suggests that the above-mentioned time-varying covariates might be confounding or mediating the relationship between baseline variables and 28- and 90-day survival. 

Regarding the secondary endpoints, it was shown that non-surgical patients exhibited a significantly higher disease severity, as indicated by general inflammatory values (i.e., serum Procalcitonin) and specific parameters for nearly every major organ system (respiratory, cardiovascular, renal, and central nervous system). However, hospital length of stay was increased in surgical sepsis patients. 

Our results suggest a potentially worse prognosis of non-surgical patients in terms of 90-day survival and sepsis-associated disease severity compared to surgical patients. These findings may be attributed to differences in baseline conditions such as comorbidities or preexisting medications (i.e., BMI, COPD, bronchial asthma, and use of statin therapy in our study population). 

These findings align with the assumption that medical sepsis patients often present with severe chronic comorbidities, whereas surgeons are less likely to operate on severely debilitated patients with high perioperative morbidity and mortality risk [23]. Additionally, early diagnosis and treatment of sepsis, which is of pivotal importance for patient outcomes, may be limited in non-surgical patients. They frequently present to the hospital at a later stage of the disease, whereas surgical patients are often extensively evaluated preoperatively, admitted to the hospital beforehand, and sepsis often manifests during their clinical stay post-intervention [8,24]. 

Furthermore, differences in the primary site of infection between the two groups should be considered as a potential influencing factor in the study’s results. Pulmonary infections were more common in non-surgical patients, whereas abdominal and surgical-site infections were more frequent in surgical patients. Previous studies have reported the prognostic role of the primary site of infection, with pulmonary infections associated with worse hospital mortality and adverse patient-centered outcomes compared to other infection types [25,26,27].

This study has limitations. It is a single-center investigation conducted at a large university medical center in Germany. The generalizability of the results would have been improved by conducting the investigation in an international multi-center design. Although the data were collected prospectively, the analyses performed were carried out in a retrospective study design. Additionally, we acknowledge the potential impact of unmeasured variables on the observed outcomes, such as the specific type and timing of surgical interventions, as well as variations in clinical management protocols. Furthermore, we must acknowledge certain limitations arising from the absence of analyses pertaining to source control and the etiology of sepsis. While our investigation focused on comparing outcomes and characteristics between surgical and non-surgical sepsis patients, we did not investigate the specific management of infection sources or identify the causative pathogens responsible for sepsis in our cohorts. Due to the inherent heterogeneity typical of sepsis cohorts, analyses of the individual reasons for intervention and patient-specific type of surgery could not be conducted in this study.

Nevertheless, we consider our findings to be important as investigations of differences in sepsis outcome between surgical and non-surgical patients are scarce. Our results underscore the need to consider patient-individual factors, such as recent surgical intervention or baseline medical condition, in sepsis treatment and care. Clear differentiation between sepsis endotypes and risk stratification based on recent surgical history are crucial in clinical decision-making and patient care.

## Figures and Tables

**Table 1:** Patient baseline characteristics with regard to surgical history.

Characteristics	All (n = 737)	Surgical (n = 582)	Non-Surgical (n = 155)	*p*-Value
Basic Conditions				
Age (years)	63 ± 15	64 ± 15	62 ± 15	0.2778
Gender (% male)	66	65	66	0.8180
Body mass index (kg/m^2^)	28 ± 7	27 ± 6	30 ± 10	0.0354
Septic Shock (%)	51	49	57	0.0841
Severity on Sepsis Onset (Day 1)				
SOFA score	10 ± 4	9 ± 4	11 ± 4	<0.001
APACHE II score	22 ± 7	21 ± 7	23 ± 7	<0.001
Use of vasopressor (%)	70	69	75	0.1024
Mechanical ventilation (%)	87	86	87	0.8278
Renal replacement therapy (%)	10	10	12	0.4636
Comorbidities (%)				
Arterial hypertension	53	53	53	0.9969
COPD	15	13	21	0.0208
Bronchial asthma	2	2	5	0.0136
Renal dysfunction	10	9	13	0.1597
NIDDM	8	9	8	0.7350
IDDM	10	10	10	0.9460
Chronic liver disease	6	6	6	0.6491
History of myocardial infarction	6	6	4	0.3010
History of stroke	5	5	6	0.5265
History of cancer	14	16	7	0.0062
Medication on Sepsis Onset (%)				
Statins	23	25	17	0.0268
Beta-blocker	37	38	31	0.1075
ACE-inhibitor	29	30	25	0.2478
Bronchodilator	10	9	14	0.0627
Diuretics	33	33	32	0.8004
Anticoagulation during the last 6 months	26	26	25	0.8094
Site of Infection (%)				
Lung	63	61	72	<0.001
Abdomen	19	22	5	
Bone or soft tissue	4	3	5	
Surgical wound	2	2	0	
Urogenital	2	2	4	
Primary bacteremia	6	5	10	
Other	4	5	4	

**Table 2:** Disease severity with regard to surgical history.

Characteristics	All (n = 737)	Surgical (n = 582)	Non-Surgical (n = 155)	*p*-Value
Sepsis Severity				
SOFA score	7.2 ± 3.7	6.9 ± 3.5	8.5 ± 4	<0.001
Days in septic shock	1 (0, 2)	0 (0, 2)	1 (0, 3)	0.0285
ICU length of stay	21 ± 16	21 ± 15	20 ± 19	0.1361
Hospital length of stay	39 ± 29	41 ± 30	32 ± 23	<0.001
Inflammatory Values				
Leukocytes (1000/µL)	13.2 ± 5	13.2 ± 5	13.3 ± 5	0.9717
C-reactive Protein (mg/L) (n = 380)	150.9 ± 85.7	151.5 ± 82.7	148.4 ± 97.7	0.3567
Procalcitonin (ng/dL) (n = 657)	1 (0.3, 3.4)	0.9 (0.3, 2.9)	1.2 (0.4, 5.1)	0.0102
Respiratory Values				
SOFA respiratory subscore	2.0 ± 0.8	1.9 ± 0.8	2.2 ± 0.8	<0.001
Patients with mechanical ventilation (%)	94	93	97	0.0807
Ventilation days/observation days (%)	68 ± 32	66 ± 32	77 ± 29	<0.001
Coagulation				
SOFA coagulation subscore	0 (0, 0.5)	0 (0, 0.54)	0.1 (0, 0.9)	0.0335
Thrombocytes (1000/µL)	292 ± 150	304 ± 153	246 ± 129	<0.001
Liver Values				
SOFA hepatic subscore	0 (0, 0.4)	0 (0, 0.4)	0 (0, 0.5)	0.3188
Bilirubin (mg/dL)	0.6 (0.4, 1.1)	0.6 (0.4, 1.1)	0.7 (0.4, 1.3)	0.3229
AST (IU/L) (n = 483)	57 (35, 112)	57 (35, 110)	54 (34, 133)	0.7879
ALT (IU/L) (n = 713)	46 (23, 92)	46 (22, 93)	43 (21, 88)	0.6174
Cardiovascular Values				
SOFA cardiovascular subscore	1.6 ± 1	1.6 ± 1	1.9 ± 1.1	<0.001
Patients with vasopressor treatment (%)	81	80	87	0.0406
Vasopressor days/observation days (%)	29 (11, 57)	28 (10, 54)	39 (18, 71)	<0.001
Central Nervous System				
SOFA central nervous system	2.1 ± 1.1	2 ± 1.1	2.2 ± 1	0.0206
Glasgow Coma Scale (GCS)	10 ± 3	10 ± 3	9 ± 3	0.0093
Renal Values				
SOFA renal subscore	0.2 (0, 1.2)	0.1 (0, 1)	0.4 (0, 2)	<0.001
Creatinine (mg/dL)	1.2 ± 0.9	1.2 ± 0.9	1.5 ± 1.1	<0.001
Urine output (mL/d)	2904 ± 1341	3027 ± 1332	2444 ± 1279	<0.001
Urine output (mL/kg/h)	1.5 ± 0.8	1.5 ± 0.8	1.2 ± 0.7	<0.001
Patients with renal replacement therapy (%)	22	20	31	0.0039
Dialysis days/observation days (%)	0 (0, 0)	0 (0, 0)	0 (0, 1)	0.0364

**Table 3:** Multivariate Cox proportional hazards regression analysis, including variables that varied as baseline.

	90-Day Mortality			28-Day Mortality		
Variables	HR	95%-CI	*p*-Value	HR	95%-CI	*p*-Value
Age	1.03	1.02–1.04	<0.001	1.03	1.02–1.05	<0.001
Male sex	1.04	0.79–1.37	0.7802	1.17	0.83–1.65	0.3683
BMI	0.99	0.97–1.01	0.2042	0.97	0.94–1	0.0243
SOFA Day 1	1.09	1.04–1.13	<0.001	1.09	1.04–1.15	<0.001
APACHE II	1.03	1–1.05	0.0629	1.03	1–1.06	0.0762
COPD	1.08	0.77–1.53	0.6544	1.06	0.69–1.62	0.7883
Bronchial asthma	0.41	0.13–1.30	0.1322	0.22	0.03–1.55	0.1271
History of cancer	1.44	1.01–2.03	0.0418	1.18	0.76–1.85	0.4622
Statin therapy	0.93	0.68–1.27	0.6435	0.79	0.54–1.16	0.2284
Surgical intervention	0.73	0.53–0.99	0.0428	0.7	0.49–1.01	0.0561

**Table 4:** Multivariate Cox proportional hazards regression analysis including time-varying covariates.

	90-Day Mortality			28-Day Mortality		
Variables	HR	95%-CI	*p*-Value	HR	95%-CI	*p*-Value
Age	1.03	1.02–1.04	<0.001	1.03	1.02–1.05	<0.001
Male sex	0.94	0.7–1.27	0.7055	0.96	0.65–1.4	0.8238
BMI	0.98	0.96–1	0.0965	0.97	0.94–1	0.0421
SOFA Day 1	0.86	0.81–0.92	<0.001	0.83	0.77–0.9	<0.001
APACHE II	0.99	0.97–1.02	0.729	1	0.96–1.04	0.9119
COPD	1.05	0.72–1.54	0.7922	0.90	0.56–1.46	0.6702
Bronchial asthma	0.59	018–1.87	0.3658	0.61	0.08–4.49	0.625
History of cancer	2.17	1.45–3.25	<0.001	1.91	1.09–3.33	0.0229
Statin therapy	1.09	0.77–1.53	0.6170	0.86	0.55–1.33	0.4863
SOFA score	1.06	0.85–1.31	0.6112	1.04	0.79–1.37	0.7644
Days in septic shock	0.96	0.92–1.01	0.1231	0.94	0.88–1	0.0494
Hospital length of stay	0.97	0.97–0.98	<0.001	0.93	0.92–0.95	<0.001
Procalcitonin	1.01	1–1.02	0.2075	1.01	1–1.02	0.1798
SOFA respiratory subscore	1.5	1.06–2.11	0.022	1.31	0.88–1.94	0.1869
Ventilation days/observation days	0.6	0.24–1.46	0.2595	0.88	0.28–2.76	0.8316
SOFA coagulation subscore	1.21	0.84–1.74	0.296	1.13	0.72–1.77	0.5837
SOFA cardiovascular subscore	3.31	1.71–6.39	<0.001	2.95	1.31–6.64	0.0092
Vasopressor days/observation days	0.18	0.03–1.22	0.0791	0.38	0.04–3.96	0.4199
SOFA central nervous system	2.36	1.65–3.38	<0.001	2.49	1.55–4.02	<0.001
SOFA renal subscore	1.7	1.19–2.41	0.0032	1.72	1.1–2.7	0.018
Urine output	0.87	0.63–1.2	0.3842	0.94	0.63–1.39	0.75
Dialysis days/observation days	0.21	0.09–0.49	<0.001	0.27	0.1–0.77	0.0143
Surgical intervention	1.08	0.78–1.5	0.6321	0.98	0.64–1.47	0.9052

## Data Availability

The datasets generated and/or analyzed during the current study are available from the corresponding author upon reasonable request.
